# A Wet-Spinning Process for Producing Carbon Nanotube/Polyvinylidene Fluoride Fibers Having Highly Consistent Electrical and Mechanical Properties

**DOI:** 10.3390/polym13224048

**Published:** 2021-11-22

**Authors:** Ki-Weon Kang, Chan-Woong Choi, Ji-Won Jin

**Affiliations:** 1 Department of Mechanical Engineering, Kunsan National University, Kunsan 54150, Korea; kwkang68@kunsan.ac.kr; 2 Strategy Planning Team, Jeonbuk Institute of Automotive Convergence Technology, Kunsan 54158, Korea; cwchoi@jiat.re.kr; 3 Green Mobility R&D Center, Jeonbuk Institute of Automotive Convergence Technology, Kunsan 54158, Korea

**Keywords:** carbon nanotube, electrical conductivity, polyvinylidene fluoride, tensile strength, wet spinning

## Abstract

Studies of polymer/carbon nanotube (CNT) fibers typically focus on optimizing the overall properties, and the effects of structural variation on these properties are ignored. Thus, we investigated the longitudinal variation in the properties of CNT/polyvinylidene fluoride (CNT/PVDF) fibers prepared by wet spinning a solution of multi-walled nanotubes, PVDF, and dimethylacetamide. To this end, materials for the CNT/PVDF fiber were selected, and a dope solution was prepared using MWNT, PVDF, and dimethylacetamide (DMAc). To consider the process parameters that would affect the performance of the CNT/PVDF fiber during the wet-spinning process using the dope solution, the initial conditions for wet spinning were selected, including bath concentration, bath temperature, drying temperature, and elongation, and the CNT/PVDF fiber was spun under the corresponding conditions. Additionally, three performance stabilization processes were proposed to improve the initial conditions for wet spinning and manufacturing the fiber. Lastly, to confirm the reliability of the CNT/PVDF fiber in all sections, tensile strength, electrical conductivity, and cross-sectional images were analyzed for the 30 m, 60 m, and 90 m sections of the fiber, and the reliability of the wet-spinning process was verified.

## 1. Introduction

Among the various nanomaterials, carbon nanotubes (CNTs) have high strength and conductivity. Therefore, they have drawn attention as ideal materials for use in coatings, fibers, and fabrics. Because of these excellent properties, CNTs have been applied in various fields, such as structural reinforcement, electrostatic shielding, and sensor materials. In particular, as a result of the superior mechanical and electrical properties of CNTs, as well as their broad range of applications, many studies into the manufacture of CNT fibers have been carried out, and it has been found that CNT-based fibers have properties that exceed those of existing high-performance fibers, such as carbon fibers [1,2,3,4,5]. CNT fibers are mainly classified into two types: neat CNT fibers, which are composed of pure CNTs, and CNT composite fibers, which are composed of CNTs dispersed in a polymer [6,7]. The manufacturing method depends on the fiber type; for example, the dry spinning method is used for neat CNT fibers, whereas the wet-spinning method is used for CNT composite fibers. In a study on the dry spinning method, Miao et al. [8] prepared CNT yarns having optimized specific strengths and specific moduli by adjusting the surface torsion angle of a high-speed rotary spin. Alvarez et al. [9] developed CNT microcables having diameters of 26.54 µm and an electrical resistance of 7 × 10^−6^ Ω·m by coating CNT fibers spinning from a CNT forest with a hydrogenated nitrile butadiene rubber polymer. In addition, Yu et al. [10] produced CNT yarns having an improved electrical conductivity, tensile strength, and Young’s modulus by adjusting the process parameters of a microcombing system. The excellent results of the above studies were obtained by controlling the dry-spinning process parameters, which improved the performance of neat CNT fibers. However, because the CNT forests used for the preparation of neat CNT fibers are prepared using chemical vapor deposition (CVD), their use is very limited, which is a severe disadvantage. To overcome this limitation, wet spinning has drawn attention. In this process, a dope solution is prepared by dispersing CNTs and polymers in a solvent, and this solution is then spun to yield CNT composite fibers. To date, there have been several studies of these composite fibers. For example, Xue et al. reported [11] electrically conductive fibers prepared by wet-spinning 40 wt% multiwalled nanotubes (MWNTs) in polyvinyl alcohol; Su et al. [12] developed CNT composite fibers by wet-spinning MWNTs in polyvinyl alcohol with gelatin; Kang et al. [13] developed electrically conductive fibers with high elasticity using the wet spinning of a silver and polyurethane mixture; and Mirbaha et al. [14] improved the fracture strength, Young’s modulus, and strain to 20% by carrying out a post-treatment process on CNT composite fibers prepared from a polyacrylonitrile (20%)/MWNT (0.75%)/dimethyl formamide (DMF) dope solution and investigated the influence of shear and elongational forces. As shown by these studies, most research into the wet spinning of CNT composite fibers has focused on the manufacturing of fibers having a single improved physical property, such as the mechanical or electrical properties. However, in the evaluation of the mechanical and electrical properties, it is difficult to confirm their homogeneity in all sections of the fiber because the sampling location and quantity of fiber specimens are not always specified. Therefore, further research into fiber manufacturing methods, specimen selection criteria, and performance evaluation methods is required.

In this study, process stabilization methods to ensure the reliability of the mechanical and electrical properties throughout all sections of CNT/PVDF fibers are proposed. To evaluate the homogeneity of the properties and structures of the CNT/PVDF fibers, the fibers were prepared using various process parameters, and the mechanical properties and electrical characteristics were evaluated in detail. MWNTs, PVDF, and dimethylacetamide (DMAc) were selected as the materials for the preparation of the CNT/PVDF fibers, and the dope solution was prepared by melting the materials together. The bath concentration, bath temperature, drying temperature, and elongation during wet spinning were selected as the key process parameters that affect the performance of the CNT/PVDF fibers. After the CNT/PVDF fibers had been prepared using the selected initial spinning conditions, their performance was evaluated by analyzing the cross-sectional morphology, tensile strength, and electrical conductivity. Based on the results, the relationship between wet spinning conditions and performance was determined, and three performance stabilization processes are proposed to improve the wet-spinning process. To confirm the reliability of these stabilization processes over all fiber sections, cross-sections of the CNT/PVDF fiber specimens prepared using different process parameters were analyzed using scanning electron microscopy of sections obtained at 30, 60, and 90 m along the fiber length, and the tensile strength and electrical conductivity were also calculated at these positions. On the basis of these results, the process reliability of the wet- spinning CNT/PVDF fibers was evaluated.

## 2. Experimental

### 2.1. Manufacture of CNT/PVDF Fibers Using Wet Spinning

#### 2.1.1. Materials

In this study, MWNTs (K-Nanos 100), which have excellent mechanical and electrical characteristics, were selected as the reinforcing agent for the CNT/PVDF fibers. PVDF (Kynar^®^761) was selected as the matrix because it is used in numerous fields, such as chemistry, electronics, pharmaceuticals, food, and paper, as a result of its excellent chemical resistance and mechanical, thermal, and electrical properties.

#### 2.1.2. Wet-Spinning Process

**Figure 1:**
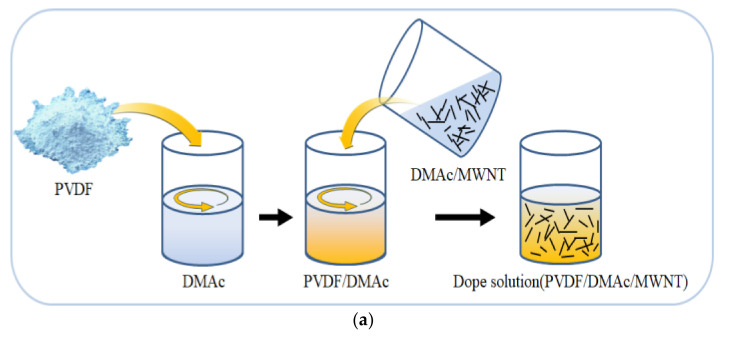

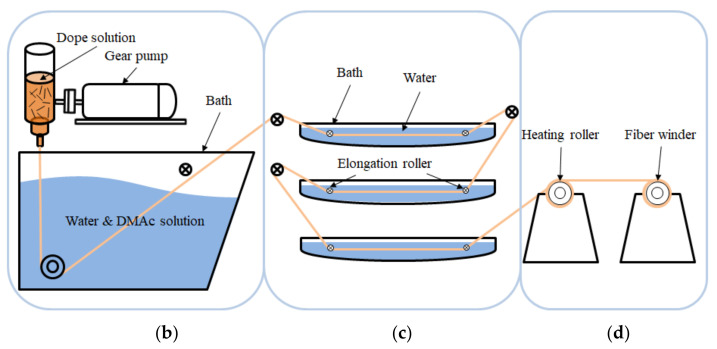
Wet-spinning process for the production of CNT/PVDF fibers. (**a**) Dope solution; (**b**) Coagulation bath; (**c**) Washing and drawing baths; (**d**) Dryer and Winding.

In this study, a wet spinning machine (DISSOL, Co., Ltd., Jeonju, Korea) was used to manufacture the CNT/PVDF fibers. Figure 1 shows the wet-spinning process, which involves four steps.

(1) Preparation of the dope solution. CNT, PVDF, and DMAc were dispersed in the molten state. Figure 1a shows the procedure for preparing the dope solution. Briefly, PVDF powder was added to a solution of DMAc in a 1:3 ratio, and the PVDF/DMAc solution was stirred for 30 min using a centrifugal stirrer. Subsequently, the PVDF/DMAc solution was added to a previously made DMAc/CNT (3 wt%) solution in a 1:4 ratio and stirred for 30 min or more using a centrifugal stirrer, thus yielding the dope solution. The composition of the final dope solution was MWNT:PVDF:DMAc = 0.6:20:79.4.
(2) Preparation of the coagulation bath. Figure 1b shows the procedure for discharging the dope solution at a spinning speed of 0.75 cc/min using a 0.05-cc gear pump and a monohole nozzle having an internal diameter of 0.66 mm. The dope solution was discharged into the coagulation bath containing aqueous DMAc, and the DMAc in the dope solution was removed into the water as a result of osmotic pressure. Thus, the dope solution solidified and crystallized to yield the fiber structure.
(3) Preparation of washing and drawing baths. Figure 1c shows the stretching of the fibers and the removal of the DMAc remaining in the dope solution, which is used to improve the orientation of the CNTs and the mechanical properties of the fibers. The bath contained water at the same temperature as the coagulation bath, and a roller was submerged in water to transfer the fibers. Then, the fibers were stretched by controlling the rotational speed of the roller.
(4) Drying and winding. Figure 1d shows the drying of the interior and exterior of the fiber using a heating roller. In this process, the fiber is heat-set by evaporating the moisture remaining in the fiber, which improves the density of the internal structure and prevents contraction. The final process is to wind the CNT/PVDF fiber spinning to a total length of 150 m using a roller.

### 2.2. Initial Process Parameters

When manufacturing CNT/PVDF fibers through wet spinning, it is very important to select the process conditions that affect the mechanical and electrical performance of the fibers. Several studies on the dependence of the fiber properties on the wet-spinning process conditions have been carried out. For example, Bell et al. [15] confirmed the effects of the shape, size, and distribution of the voids formed inside the fibers during wet spinning on the mechanical properties, as well as the effects of the diffusion rate of the solvent and solidifying agent. The solidification conditions, such as bath concentration and bath temperature, were reported to be the key parameters determining the diffusion rates. Mai et al. [16] confirmed that the elongation of fibers could affect the dispersion of MWNTs inside the polymer during the manufacturing of polyimide-66/MWNT fibers. Kim et al. [17] manufactured cellulose nanofibers through wet spinning and confirmed that the drying temperature was correlated with the formation of voids inside the fiber. Based on the above studies, in this study, the bath concentration, bath temperature, drying temperature, and elongation were selected as the process parameters affecting the fiber performance. Thus, the initial process parameters to manufacture the CNT/PVDF fibers were the bath concentration (60%), bath temperature (60 °C), drying temperature (120 °C), and elongation (200%), and the effects of these process parameters on the fiber properties were investigated.

### 2.3. Performance Evaluation of Wet-Spinning CNT/PVDF Fibers

Three methods are proposed to evaluate the performance of the CNT/PVDF fibers. The first method is based on the analysis of the structural density inside the CNT/PVDF fiber and the distribution of CNTs and voids, which affect the mechanical properties. For this method, cross-sectional images of the fibers were captured by scanning electron microscopy (SEM, SU82220, HITACHI). To confirm the homogeneity of the fiber along its length, three sections at 30, 60, and 90 m along the length of the fibers were used.

**Figure 2:**
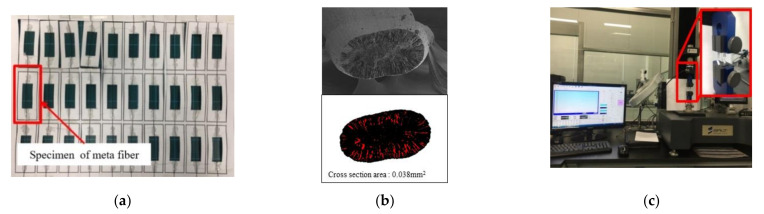
Process of CNT/PVDF fiber tensile tests. (**a**) Preparation of CNT/PVDF fiber; (**b**) Measurement of the CNT/PVDF fiber cross-sectional area; (**c**) CNT/PVDF fiber tensile tests.

The second method is tensile testing, which reveals the mechanical properties of the CNT/PVDF fibers. These tests were carried out in compliance with the ASTM D3379 standard [18]. As shown in Figure 2a, to prepare tensile specimens of the CNT/PVDF fibers, both ends of a fiber were fixed to a paper guide with an adhesive considering a gauge length of 25 mm. To calculate the tensile strength, the cross-sectional area of the fiber used in the tensile tests was measured using image analysis of the SEM micrographs (Figure 2b) [19]. Image J [20] was used for this image analysis. The actual area of the fiber was calculated based on the area colored in black in Figure 2b, which excludes blank spaces and voids, which are colored in white and red, respectively. The tensile strength of the CNT/PVDF fibers was assessed using a universal testing machine (UTM, ST-1000, SALT Co., Ltd., Incheon, Korea) (Figure 2c) at a draw rate of 2 mm/min under atmospheric conditions at room temperature.

**Figure 3:**
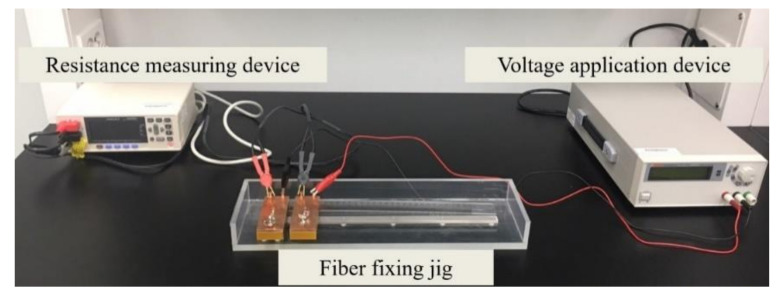
Device for measuring the fiber resistance.

The last method involves the measurement of the electrical conductivity of the CNT/PVDF fibers. For these tests, a four-point probe offsetting the surface resistance is generally used [21,22]. However, in this study, a fiber resistance measurement system minimizing the contact resistance was used, as shown in Figure 3, which allowed us to consider conditions under which no voltage was applied, as used in industrial fields. The system consists of a resistance measurement instrument (RM3544, HIOKI) for measuring the micro-resistance, a voltage application device (OPE-305, ODA Technologies, Incheon, Korean), and a jig to fix the fibers depending on their length. The tests followed the IEC 60,093 standard [23] for determining electrical conductivity data. After mounting a fiber on the fixing jig at a gauge length of 1 cm under atmospheric conditions at room temperature, a potential of 10 V was applied for 10 s, and the resistance was measured with the voltage shut off. Then, the calculated resistance was converted to electrical conductivity.

## 3. Results and Discussion

### 3.1. Performance Evaluation

**Figure 4:**
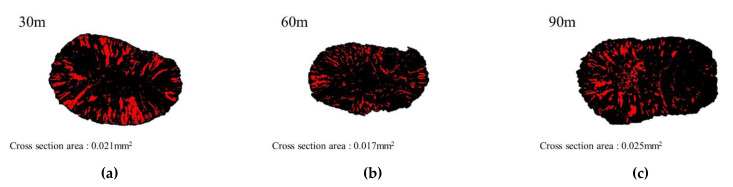
Cross-sectional area of initial condition CNT/PVDF fibers. (**a**) Specimen 30 m; (**b**) Specimen 60 m; (**c**) Specimen 90 m.

**Figure 5:**
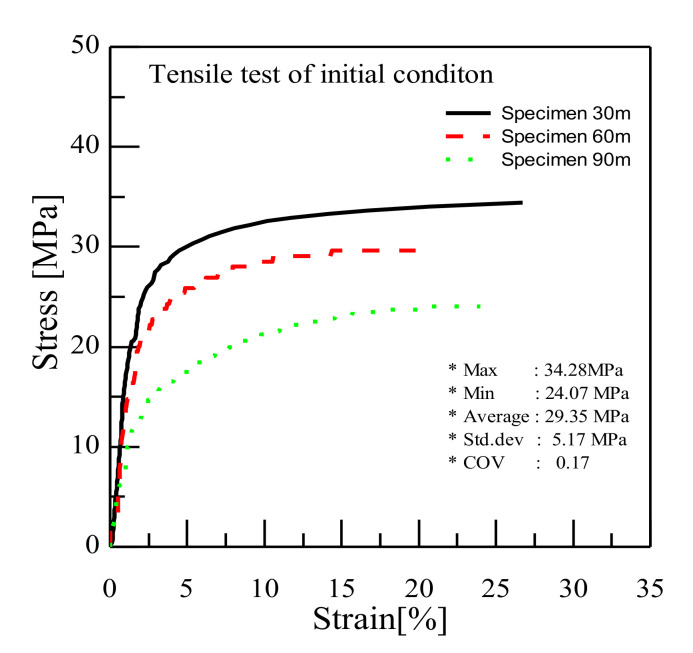
Results of the tensile test for initial condition CNT/PVDF fibers.

**Figure 6:**
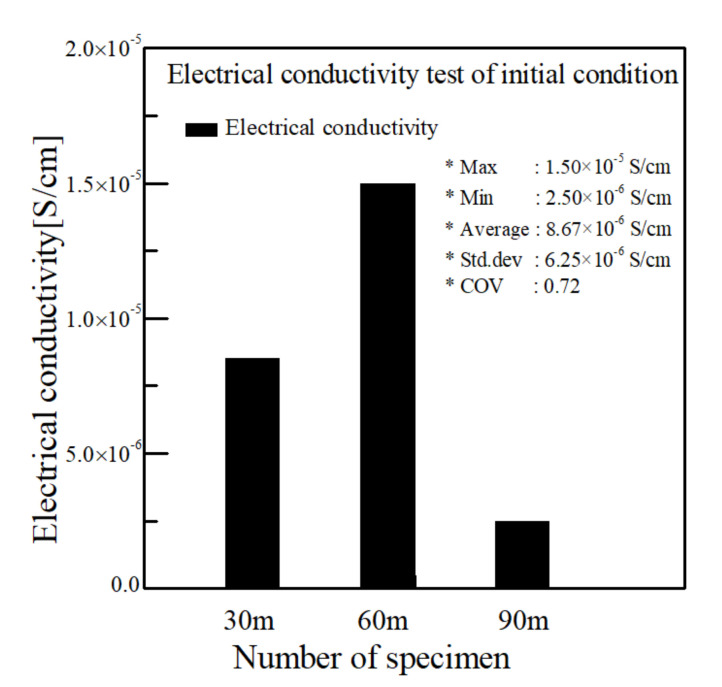
This is a figure. Schemes follow the same formatting.

**Figure 7:**
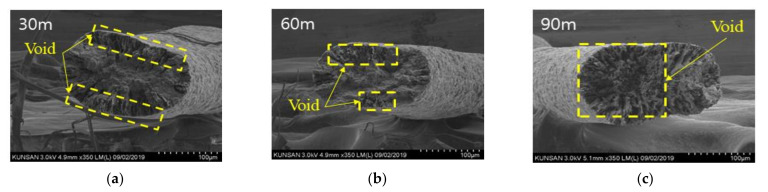
SEM images of the cross section for the initial condition. (**a**) Specimen 30 m; (**b**) Specimen 60 m; (**c**) Specimen 90 m.

CNT/PVDF fibers were prepared using the initial processing parameters and conditions described in Section 2.2, and their performance was evaluated using the three methods described in Section 2.3. First, to analyze the internal structural density and the distribution of CNTs and voids, SEM cross-sectional images of the CNT/PVDF fibers were obtained at 30, 60, and 90 m, along the fiber length (Figure 4). As shown in the figure, the fiber and void distributions varied between the sampled regions, confirming that there is significant variation in the cross-sectional area of the fibers along their length. These results are reflected in the tensile test results for each section, and the corresponding stress–strain curves are shown in Figure 5. The tensile strengths for the 30, 60, and 90 m sections were calculated to be 34.39, 29.59, and 24.07 MPa, respectively. Figure 6 shows the electrical conductivities of the CNT/PVDF fibers, which were found to be 8.50 × 10^−6^, 1.50 × 10^−5^, and 2.50 × 10^−6^ S/cm, respectively. Based on these results, the variation in the cross-sectional structure, tensile strength, and electrical conductivity in the longitudinal direction of the CNT/PVDF fibers was confirmed. To understand this variation, the cross-sections of the CNT/PVDF fibers were magnified 350 times (Figure 7), and further image analysis was carried out. As seen in this figure, more voids were present in the outer regions than in the fiber center in the 30 m section (Figure 7a), and this resulted in the highest tensile strength among the measured sections. For the sample obtained at 60 m (Figure 7b), the voids were distributed in the outer layer of the fiber only in certain areas, and this sample had the second-highest tensile strength and the highest electrical conductivity. For the sample obtained at 90 m (Figure 7c), the voids were distributed throughout the entire fiber cross-section, and this sample had the lowest tensile strength and electrical conductivity. These results suggest that the size and location of the voids inside the fiber affect the tensile strength and electrical conductivity. Therefore, it is necessary to reduce voids through process stabilization to ensure the manufacture of CNT/PVDF fibers having a constant tensile strength and electrical conductivity along the fiber length.

### 3.2. Process Stabilization to Ensure the Reliability and Continuity of CNT/PVDF Fibers

The formation of voids in the CNT/PVDF fibers is the key factor affecting their tensile strengths and electrical conductivities. Therefore, techniques for reducing the formation of voids inside the fibers are key to the manufacture of reliable fibers. Thus, next, we added three processes to the production of the CNT/PVDF fibers to reduce the formation of voids. First, the dope solution was stored at 40 °C for 24 h to remove the voids. Secondly, in the coagulation bath, the dope solution was placed in a cylinder at room temperature, and air was introduced at a pressure of 0.4 MPa for 1 h to remove any remaining voids inside the dope solution; subsequently, the fibers were pressed at a pressure of 0.4 MPa. Thirdly, the wound CNT/PVDF fibers were dried at room temperature for five days to remove any moisture remaining inside the fiber. To determine the effects of the three processes on the reduction of voids inside the fiber and the resulting improvement in performance reliability, various process parameters were selected for the manufacture and evaluation of the fibers.

### 3.3. Evaluation of Performance-Stabilized Fibers

As discussed in Section 3.1, owing to the irregular void distribution inside the CNT/PVDF fibers, there is variation in the physical properties in the longitudinal direction of the fiber. Therefore, we evaluated the effects of changing the wet-spinning conditions on the properties of all sections of the fibers, and the tested conditions are shown in Table 1. Subsequently, cross-sectional SEM images were obtained, and the tensile strengths and electrical conductivities of the different fibers were assessed.

**Figure 8:**
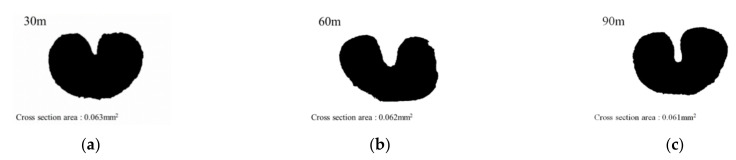
Cross section area of case 1 ($x_{1}$:$x_{2}$:$x_{3}$:$x_{4}$ = 40:60:120:200). (**a**) Specimen 30 m; (**b**) Specimen 60 m; (**c**) Specimen 90 m.

**Figure 9:**
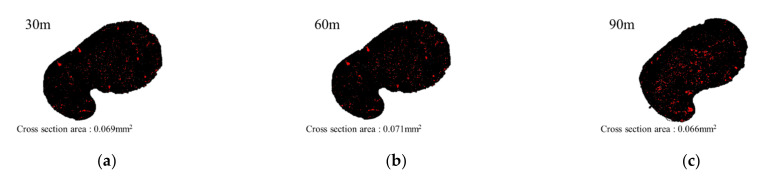
Cross section area of case 2 ($x_{1}$:$x_{2}$:$x_{3}$:$x_{4}$ = 60:60:120:200). (**a**) Specimen 30 m; (**b**) Specimen 60 m; (**c**) Specimen 90 m.

Fist, SEM images of the 30, 60, and 90 m fiber sections for Cases 1 and 2 in Table 2 were analyzed (Figure 8 and Figure 9) to calculate the cross-sectional area of the spinning fiber and evaluate their structures. Table 2 shows the cross-sectional results for Cases 1–8. The cross-sectional areas of the CNT/PVDF fibers showed little variability, as shown by the coefficients of variation (COV) of the 30, 60, and 90 m sections of 0.011–0.074. Therefore, it was confirmed that the additional post-treatment processes increased the manufacturing stability of the CNT/PVDF fibers.

**Figure 10:**
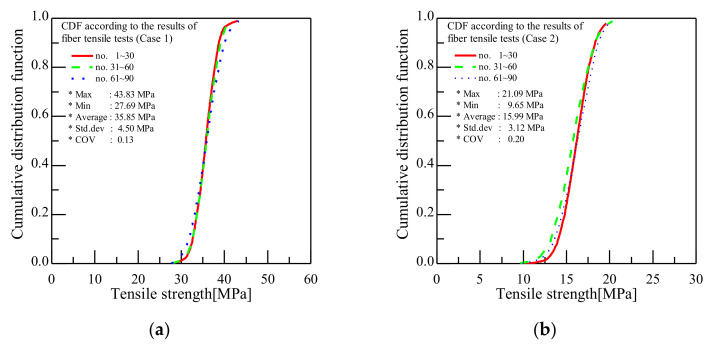
Cumulative distribution functions for tensile strength. (**a**) Case 1 ($x_{1}$:$x_{2}$:$x_{3}$:$x_{4}$ = 40:60:120:200); (**b**) Case 2 ($x_{1}$:$x_{2}$:$x_{3}$:$x_{4}$ = 60:60:120:200).

Next, we examined the mechanical properties of the fibers in all sections. For this, 30 specimens were sampled at 30, 60, and 90 m and subjected to tensile tests. Table 3 and Table 4 show the tensile strength results obtained after a total of 90 tensile tests of samples prepared using the conditions labeled Cases 1 and 2 in Table 1. The tensile strength COV indicates little variation, ranging from 0.11 to 0.14 for Case 1 and from 0.19 to 0.21 for Case 2. Table 5 summarizes the tensile test results of the fibers with respect to the selected process parameters. Although the magnitude of the COV varied between cases, the COVs for the different sampled sections (30, 60, and 90 m) remained constant for Cases 1–8. These results confirm the continuity of the mechanical properties along the fiber length and suggest that the bath concentration, bath temperature, drying temperature, and elongation affect the tensile strength. In addition, the tensile test results of Cases 1 and 2 are expressed as cumulative distribution functions (CDFs). Figure 10a shows the CDF diagram for Case 1, which has a COV of 0.13, confirming that the tensile strengths in all tested sections are constant. Figure 10b shows the CDF diagram for Case 2. Although the tensile strength was found to vary with 9.65 MPa in specimens 31–60, the COV was only 0.20, indicating that the trend in the CDF diagram was constant. Thus, the three CDF diagrams, which represent the average tensile strength of the 30 specimens from each of the 30, 60, and 90 m sections of the fibers, mostly overlapped, indicating minimal variation in the tensile strength in the tested sections of the fibers.

**Figure 11:**
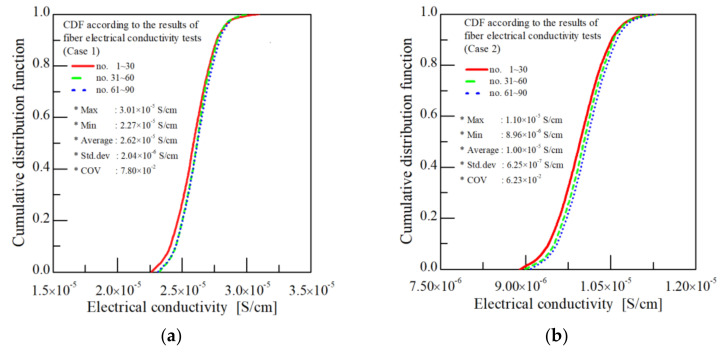
Cumulative distribution functions for electrical conductivity. (**a**) Case 1 ($x_{1}$:$x_{2}$:$x_{3}$:$x_{4}$ = 40:60:120:200); (**b**) Case 2($x_{1}$:$x_{2}$:$x_{3}$:$x_{4}$ = 60:60:120:200).

Next, the consistency in the electrical properties of the CNT/PVDF fiber in the longitudinal direction was analyzed, and, as before, 30 specimens were sampled from the 30, 60, and 90 m sections. Table 6 and Table 7 show the corresponding results for Cases 1 and 2 (see Table 1). The electrical conductivity COV values for the 30, 60, and 90 m sections for Cases 1 and 2 ranged from 7.41 × 10^−2^ to 8.23 × 10^−2^ and 5.55 × 10^−2^ to 7.03 × 10^−2^, respectively, indicating little variation. Table 8 summarizes the electrical conductivity results for the fiber depending on the selected process parameters. Although the magnitude of the COV varied between cases, the COV remained constant for all sections for Cases 1–8. These results confirm that the electrical properties of the fibers were consistent along the fiber length, and also suggest that the bath concentration, bath temperature, drying temperature, and elongation affect the electrical conductivity. As for the tensile tests, the electrical conductivities of the fibers prepared using the conditions in Cases 1 and 2 in Table 1 are expressed as CDFs in Figure 11. As shown in Figure 11a, the three CDF diagrams for Case 1, which represent the average of the 30 electrical conductivity measurements, mostly overlap, indicating minimal variance in electrical conductivity across the sections of the fiber.

**Figure 12:**
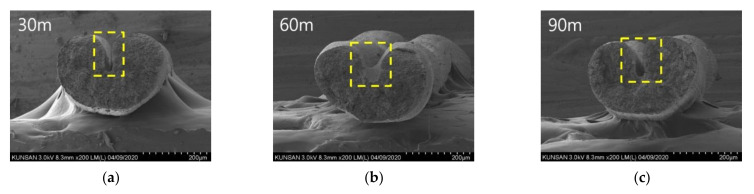
SEM images of case 1 ($x_{1}$:$x_{2}$:$x_{3}$:$x_{4}$ = 40:60:120:200). (**a**) Specimen 30 m; (**b**) Specimen 60 m; (**c**) Specimen 90 m.

**Figure 13:**
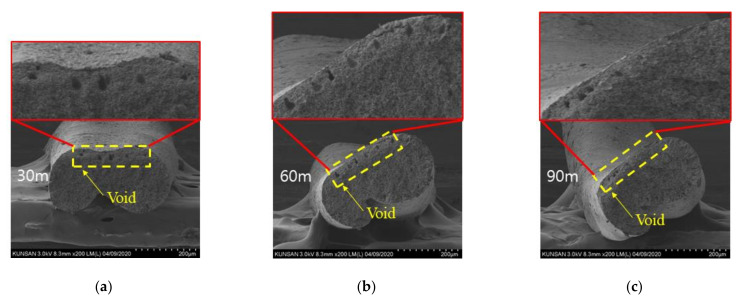
SEM images of case 2 ($x_{1}$:$x_{2}$:$x_{3}$:$x_{4}$ = 60:60:120:200). (**a**) Specimen 30 m; (**b**) Specimen 60 m; (**c**) Specimen 90 m.

In addition, the variation in the cross-sectional shape of the CNT/PVDF fibers was analyzed using SEM. Figure 12 and Figure 13 show the SEM images of the cross-sections of the 30, 60, and 90 m sections for Cases 1 and 2. The SEM image for Case 1 in Figure 12 confirms a constant shape in each section, whereas that for Case 2 in Figure 13 demonstrates that the voids were constant throughout each fiber section.

Finally, the average tensile strength and the average electrical conductivity were analyzed as a function of the void distribution, and these properties were found to be the highest for fibers prepared using Case 1, which had no voids. The process parameters of Case 1 and Case 2 were identical except for the bath concentration, which was 20% higher in Case 2 than in Case 1. Therefore, we believe that, in Case 2, some of the fiber did not solidify, thus creating voids inside the fiber and reducing the tensile strength.

## 4. Conclusions

In this study, performance stabilization processes to ensure that the electrical and mechanical properties of CNT/PVDF fibers are homogenous with respect to the fiber length were developed. Crucially, a wet-spinning procedure for the manufacture of CNT/PVDF fibers was optimized, and the process parameters (bath temperature, bath concentration, drying temperature, and elongation) that could affect the fiber performance were investigated. In addition, tests of the tensile properties, electrical conductivity, and fiber morphology were carried out to assess the performance of the CNT/PVDF fibers. The results indicate that the fiber shape and voids were constant across the 30, 60, and 90 m sections of the fibers prepared using the conditions in Cases 1–8. In addition, the size of the voids inside the fiber were found to be inversely proportional to the tensile strength and electrical conductivity, whereas the CNTs inside the CNT/PVDF fiber were evenly distributed, thus confirming the reliability of our optimized wet-spinning process. As a result of these analyses, the main cause affecting the performance of the CNT/PVDF fiber was found to be the voids formed inside the fiber. Therefore, we propose that a wet-spinning process that yields minimal voids inside the fibers is key to ensuring the production of CNT/PCDF fibers having highly reliable electrical and mechanical properties.

## Figures and Tables

**Table 1:** Conditions of wet spinning.

Case	Bath Concentration *x*_1_, (%)	Bath Temperature *x*_2_, (°C)	Drying Temperature *x*_3_, (°C)	Elongation *x*_4_, (%)
1	40	60	120	200
2	60	60	120	200
3	40	40	100	200
4	40	60	100	200
5	40	40	100	400
6	40	40	120	400
7	50	50	110	100
8	50	50	110	300

**Table 2:** Distribution of the cross-sectional area for CNT/PVDF fiber conditions.

Position (m)	Area (mm^2^)							
	Case 1	Case 2	Case 3	Case 4	Case 5	Case 6	Case 7	Case 8
30	0.063	0.069	0.052	0.061	0.029	0.024	0.098	0.051
60	0.062	0.071	0.053	0.067	0.030	0.025	0.100	0.050
90	0.061	0.066	0.053	0.060	0.029	0.028	0.100	0.046
Total Average (mm^2^)	0.062	0.069	0.052	0.063	0.030	0.026	0.099	0.049
Std. dev (mm^2^)	0.001	0.003	0.001	0.004	0.000	0.002	0.001	0.003
COV	0.014	0.037	0.013	0.058	0.012	0.074	0.011	0.054

**Table 3:** Results of tensile tests for Case 1.

No.	Tensile Strength (MPa)	No.	Tensile Strength (MPa)	No.	Tensile Strength (MPa)
1	33.20	31	36.52	61	40.92
2	31.85	32	33.74	62	43.83
3	28.43	33	37.19	63	30.89
4	39.04	34	40.23	64	35.14
5	30.68	35	40.67	65	30.90
⋮	⋮	⋮	⋮	⋮	⋮
30	40.44	60	31.79	90	35.91
Max	43.41	Max	43.83	Max	43.83
Min	27.69	Min	29.86	Min	27.69
Average	36.19	Average	35.76	Average	35.60
Std. dev	4.78	Std. dev	3.90	Std. dev	4.89
COV	0.13	COV	0.11	COV	0.14
Total max(No.1~No.90) (MPa)			44.83		
Total min(No.1~No.90) (MPa)			27.69		
Total average(No.1~No.90) (MPa)			35.85		
Total std. dev(No.1~No.90) (MPa)			4.50		
Total COV(No.1~No.90)			0.13		

**Table 4:** Results of tensile tests for Case 2.

No.	Tensile Strength (MPa)	No.	Tensile Strength (MPa)	No.	Tensile Strength (MPa)
1	13.10	31	13.10	61	18.79
2	15.34	32	19.33	62	18.64
3	13.89	33	12.37	63	12.29
4	16.20	34	20.53	64	13.64
5	18.92	35	20.17	65	12.99
⋮	⋮	⋮	⋮	⋮	⋮
30	19.50	60	13.99	90	18.54
Max	21.09	Max	20.88	Max	20.88
Min	10.08	Min	9.65	Min	11.16
Average	15.87	Average	16.23	Average	15.88
Std. dev	3.00	Std. dev	3.13	Std. dev	3.32
COV	0.19	COV	0.19	COV	0.21
Total max(No.1~No.90) (MPa)			21.09		
Total min(No.1~No.90) (MPa)			9.65		
Total average(No.1~No.90) (MPa)			15.99		
Total std. dev(No.1~No.90) (MPa)			3.12		
Total COV(No.1~No.90)			0.20		

**Table 5:** Results of tensile tests for Cases 1~8.

Case	Tensile Strength				
	Max (MPa)	Min (MPa)	Average (MPa)	Std. Dev (MPa)	COV
1	43.83	27.69	35.85	4.50	0.13
2	21.09	9.65	15.99	3.12	0.20
3	20.08	15.93	17.94	1.05	0.06
4	41.64	26.92	34.16	4.18	0.12
5	18.28	12.65	15.33	1.62	0.11
6	30.10	22.06	26.48	2.21	0.08
7	17.33	14.30	15.88	0.78	0.05
8	31.06	19.06	25.02	3.66	0.15

**Table 6:** Results of the electrical conductivity test for Case 1.

No.	Electrical Conductivity (S/cm)	No.	Electrical Conductivity (S/cm)	No.	Electrical Conductivity (S/cm)
1	2.47 × 10^−5^	31	2.39 × 10^−5^	61	2.44 × 10^−5^
2	3.01 × 10^−5^	32	2.97 × 10^−5^	62	2.37 × 10^−5^
3	2.81 × 10^−5^	33	2.59 × 10^−5^	63	2.69 × 10^−5^
4	2.77 × 10^−5^	34	2.28 × 10^−5^	64	2.68 × 10^−5^
5	2.79 × 10^−5^	35	2.35 × 10^−5^	65	2.91 × 10^−5^
⋮	⋮	⋮	⋮	⋮	⋮
30	2.85 × 10^−5^	60	2.77 × 10^−5^	90	2.39 × 10^−5^
Max	3.01 × 10^−5^	Max	2.98 × 10^−5^	Max	2.98 × 10^−5^
Min	2.30 × 10^−5^	Min	2.28 × 10^−5^	Min	2.27 × 10^−5^
Average	2.59 × 10^−5^	Average	2.64 × 10^−5^	Average	2.62 × 10^−5^
Std. dev	2.13 × 10^−6^	Std. dev	2.09 × 10^−6^	Std. dev	1.94 × 10^−6^
COV	8.23 × 10^−2^	COV	7.92 × 10^−2^	COV	7.41 × 10^−2^
Total max(No.1~No.90) (S/cm)			3.01 × 10^−5^		
Total min(No.1~No.90) (S/cm)			2.27 × 10^−5^		
Total average(No.1~No.90) (S/cm)			2.62 × 10^−5^		
Total std. dev(No.1~No.90) (S/cm)			2.04 × 10^−6^		
Total COV(No.1~No.90)			7.80 × 10^−2^		

**Table 7:** Results of the electrical conductivity test for Case 2.

No.	Electrical Conductivity (S/cm)	No.	Electrical Conductivity (S/cm)	No.	Electrical Conductivity (S/cm)
1	1.05 × 10^−5^	31	9.71 × 10^−6^	61	1.08 × 10^−5^
2	8.96 × 10^−6^	32	1.01 × 10^−5^	62	9.80 × 10^−6^
3	9.49 × 10^−6^	33	1.06 × 10^−5^	63	9.44 × 10^−6^
4	1.08 × 10^−6^	34	1.03 × 10^−5^	64	9.72 × 10^−6^
5	1.01 × 10^−6^	35	9.67 × 10^−6^	65	1.01 × 10^−5^
⋮	⋮	⋮	⋮	⋮	⋮
30	1.09 × 10^−5^	60	9.67 × 10^−6^	90	1.05 × 10^−5^
Max	1.10 × 10^−5^	Max	1.10 × 10^−5^	Max	1.09 × 10^−5^
Min	8.96 × 10^−6^	Min	8.97 × 10^−6^	Min	9.01 × 10^−6^
Average	1.00 × 10^−5^	Average	9.95 × 10^−6^	Average	1.00 × 10^−5^
Std. dev	7.06 × 10^−7^	Std. dev	6.21 × 10^−7^	Std. dev	5.58 × 10^−7^
COV	7.03 × 10^−2^	COV	6.23 × 10^−2^	COV	5.55 × 10^−2^
Total max(No.1~No.90) (S/cm)			1.10 × 10^−5^		
Total min(No.1~No.90) (S/cm)			8.96 × 10^−6^		
Total average(No.1~No.90) (S/cm)			1.00 × 10^−5^		
Total std. dev(No.1~No.90) (S/cm)			6.25 × 10^−7^		
Total COV(No.1~No.90)			6.24 × 10^−2^		

**Table 8:** Results of electrical conductivity tests for Cases 1~8.

Case	Electrical Conductivity				
	Max (S/cm)	Min (S/cm)	Average (S/cm)	Std. dev (S/cm)	COV
1	3.01 × 10^−5^	2.27 × 10^−5^	2.62 × 10^−5^	2.04 × 10^−6^	7.80 × 10^−2^
2	1.10 × 10^−5^	8.96 × 10^−6^	1.00 × 10^−5^	6.25 × 10^−7^	6.24 × 10^−2^
3	1.09 × 10^−5^	8.53 × 10^−6^	9.64 × 10^−6^	6.50 × 10^−7^	6.74 × 10^−2^
4	1.74 × 10^−5^	1.30 × 10^−5^	1.51 × 10^−5^	1.15 × 10^−6^	7.61 × 10^−2^
5	1.56 × 10^−5^	1.10 × 10^−5^	1.31 × 10^−5^	1.33 × 10^−6^	1.02 × 10^−1^
6	3.66 × 10^−5^	2.51 × 10^−5^	3.04 × 10^−5^	3.33 × 10^−6^	1.10 × 10^−1^
7	4.36 × 10^−5^	3.43 × 10^−5^	3.91 × 10^−5^	2.90 × 10^−6^	7.41 × 10^−2^
8	1.26 × 10^−5^	9.72 × 10^−6^	1.11 × 10^−5^	9.03 × 10^−7^	8.11 × 10^−2^

## Data Availability

The data presented in this study are available on request from the corresponding author. The data are not publicly available due to privacy of this research.
